# TRPV1 activation power can switch an action mode for its polypeptide ligands

**DOI:** 10.1371/journal.pone.0177077

**Published:** 2017-05-05

**Authors:** Maxim V. Nikolaev, Natalia A. Dorofeeva, Margarita S. Komarova, Yuliya V. Korolkova, Yaroslav A. Andreev, Irina V. Mosharova, Eugene V. Grishin, Denis B. Tikhonov, Sergey A. Kozlov

**Affiliations:** 1 I.M.Sechenov Institute of Evolutionary Physiology and Biochemistry RAS, St.Petersburg, Russia; 2 Shemyakin-Ovchinnikov Institute of Bioorganic Chemistry, RAS, Moscow, Russia; 3 Sechenov First Moscow State Medical University, Institute of Molecular Medicine, Moscow, Russia; Indiana University School of Medicine, UNITED STATES

## Abstract

TRPV1 (vanilloid) receptors are activated by different types of stimuli including capsaicin, acidification and heat. Various ligands demonstrate stimulus-dependent action on TRPV1. In the present work we studied the action of polypeptides isolated from sea anemone *Heteractis crispa* (APHC1, APHC2 and APHC3) on rat TRPV1 receptors stably expressed in CHO cells using electrophysiological recordings, fluorescent Ca^2+^ measurements and molecular modeling. The APHCs potentiated TRPV1 responses to low (3–300 nM) concentrations of capsaicin but inhibited responses to high (>3.0 μM) concentrations. The activity-dependent action was also found for TRPV1 responses to 2APB and acidification. Thus the action mode of APHCs is bimodal and depended on the activation stimuli strength—potentiation of low-amplitude responses and no effect/inhibition of high-amplitude responses. The double-gate model of TRPV1 activation suggests that APHC-polypeptides may stabilize an intermediate state during the receptor activation. Molecular modeling revealed putative binding site at the outer loops of TRPV1. Binding to this site can directly affect activation by protons and can be allosterically coupled with capsaicin site. The results are important for further investigations of both TRPV1 and its ligands for potential therapeutic use.

## Introduction

TRPV1 is the founding member of a TRP channel sub-family, which has six members divided into two groups: thermo-sensitive, non-selective ion channels (TRPV1–TRPV4) and channels highly selective for Ca^2+^ ions (TRPV5-TRPV6) (see [1] for review). TRPV1 is known as the capsaicin receptor or vanilloid receptor 1. It is the most thoroughly studied channel among TRPVs. TRPV1 mediate pronounced cation influx and have weak selectivity to Ca^2+^. Various activation stimuli including capsaicin, resiniferatoxin, heat, H^+^, endocannabinoid lipids such as anandamide, eicosanoids, and 2APB were identified as current agonists of TRPV1 [2–6].

Since elaboration of TRPV1 pharmacology has obvious perspectives related to the potential treatment of pain and inflammation, many compounds affecting TRPV1 activation have been characterized [7–9]. Some TRPV1 ligands have distinct effects on different modes of channel activation (heat, protons or chemical ligands). For example, compound JYL-1421 blocks responses to capsaicin but not to heat or protons [10]. This action profile was obtained on rat TRPV1, but could not be shown for human or monkey TRPV1 [10, 11]. Well-known TRPV1 antagonist capsazepine also demonstrates specificity of effects; it is ineffective as an antagonist of proton-induced response in rat TRPV1, while heat and capsaicin responses are blocked at low capsazepine concentrations [11]. Another potent and selective TRPV1 modulator, AMG8562, blocks capsaicin activation, does not affect heat activation, and potentiates proton activation of rat TRPV1 [8]. It blocks both heat and capsaicin activation of human TRPV1 but acts as a partial antagonist of proton activation [11]. The origin of these dramatically different selectivity profiles remains unknown.

The attempts to reveal the molecular determinants of different TRPV1 gating modes have led to identification of several clusters of amino acid residues involved in channel activation. The capsaicin site was found by comparison of capsaicin-sensitive rat TRPV1 and its chicken homolog, which is capsaicin-insensitive [12]. Critical residues were found in the MT2-TM3 region located on the lipid-facing periphery of the tetrameric channel complex [13, 14]. In contrast, residues controlling channel activation by temperature or protons are located at the extracellular receptor part and belong to overlapping sites [15–19]. Complex allosteric interrelations between these differently localized activation sites remains unclear.

Small lipophilic drugs can potentially reach their binding sites in different parts of a receptor, which complicates the study of mechanisms of action. Binding sites for membrane-impermeable peptide modulators should be localized at the outside surface of a channel protein. There are a few examples of polypeptides affecting TRPV1 channels. Peptide agonists of TRPV1 are vanillotoxins (VaTx1-3) isolated from the venom of tarantula *Psalmopoeus cambridgei* [20] the bivalent toxin DkTx from tiger tarantula *Ornithoctonus huwena* [21] and RhTx from the venom of the Chinese red-headed centipede [22]. They cause pain behavior when injected in mice. Polypeptides APHC1 and APHC3 from the sea anemone *Heteractis crispa* were characterized as inhibitors of capsaicin-induced currents in TRPV1-expressing cells [23, 24]. They produce significant analgesic effects associated with TRPV1 inhibition in different animal models at doses of 0.01–0.1 mg/kg [23, 25–27]. In the present work we used sea anemone *H*. *crispa* polypeptides APHC1-3 for analysis of their mechanism of action on TRPV1 by electrophysiological, Ca^2+^ imaging and modeling approaches.

Recently, the atomic-scale structures of TRPV1 in different functional states were published [28, 29]. Channel structures in the presence of vanillotoxin and capsaicin provide clues to understanding the complex gating mechanism of TRPV1. The channel has two gate regions: in the selectivity filter and in the inner pore. The dual-gate model of TRPV1 activation provides background for kinetic modeling of ligand action on TRPV1. Particularly, the presence of two distinct intermediate states (with only the outer or inner gate open) potentially helps to explain the qualitatively different action of some ligands on different activation modes of TRPV1. In the present work we employed these recent findings to analyze the experimental data on action of APHC polypeptides on TRPV1 channels.

## Materials and methods

### Production of recombinant polypeptides

Polypeptides APHC1-3 were produced as previously described [24]. DNA fragments encoding the polypeptides were cloned into the expression vector pET32b+ (Novagen, USA). *Escherichia coli* BL21 (DE3) cells expressing thioredoxin fusions of polypeptides were cultured overnight at 25°C, harvested, ultrasonicated in a buffer for metal-affinity chromatography, and centrifuged to remove all insoluble particles. Fusion proteins were purified using a TALON Superflow Metal Affinity Resin (Clontech, USA) and subjected to CNBr cleavage, as described [30]. The recombinant polypeptides were purified on a reverse-phase column Jupiter C_5_ (Phenomenex, USA) 250×10 mm. The target polypeptides’ purity was verified by MALDI-TOF mass-spectrometry.

### Fluo-4 based intracellular calcium assay

CHO (Chinese hamster ovary) cells were obtained from Evrogen company (http://evrogen.ru). The CHO cell lines stably expressing rat TRPV1 was generated using T-Rex System (Invitrogen) according to the manufacturer’s instructions and fluorescent assays were performed using NOVOstar (BMG LABTECH, Germany) as described [31]. CHO cells stably expressing rat TRPV1 were seeded into black-walled, clear-bottomed 96-well plates at a density of 75,000 cells per well (complete media without antibiotics and containing 1 μg/ml tetracycline to induce channel expression) and were cultured overnight at 37°C. The cells were then loaded with the cytoplasmic calcium indicator Fluo-4AM using Fluo-4 Direct^™^ Calcium Assay Kits (Invitrogen) and incubated in the dark at 37°C for 60 minutes and then at 25°C for 60 minutes. The buffer alone (control) or APHC-polypeptides were added to the cells. Changes in cell fluorescence (λex = 485 nM, λem = 520 nM) were monitored before and after the addition of TRPV1 agonist (capsaicin or 2APB). The measurements were performed at pH 7.4 and 25°C.

### Electrophysiology

Recordings were made by a patch-clamp technique in whole-cell configuration using CHO cells stably expressing rat TRPV1. Whole-cell currents were recorded and digitized by Instru TECH LIH 8+8 data acquisition system (HEKA, Germany) at -80 mV holding voltage using EPC-8 (HEKA, Germany) amplifier, filtered at 5 kHz, sampled at 10kHz and stored. The external solution contained in mM: CaCl_2_ 2.5, KCl 5, NaCl 143, D-glucose 18, HEPES 10, pH 7.35. Patch pipettes (2–5 MΩ resistance) were prepared by puller model P-97 (Sutter Instruments, USA). Pipette solution contained in mM: CsF 100, CsCl 40, NaCl 5, CaCl_2_ 0.5, EGTA 5, HEPES 10, pH 7.2. Series resistance of about 20 MΩ was compensated by 70–80% and monitored during experiments. Drugs were applied using the ALA-VM8 (ALA Scientific Instruments, USA) perfusion system under computer control. All experiments were performed at 22°C.

### Molecular modeling

All calculations were performed using the ZMM program (www.ZMMsoft.com). Non-bonded interactions were calculated with the AMBER force field [32, 33] and a cutoff distance of 8 Å. Electrostatic interactions were calculated by using the distance- and environment-dependent dielectric function [34]. Bond lengths and bond angles were kept rigid during the calculations. The Monte Carlo Minimization (MCM) method [35] was used to optimize the models. All side chain torsions were randomly sampled during the MCM trajectories. Both side chains and backbones were flexible during energy minimizations. MCM of each model was performed until 1,000 consecutive energy minimizations did not decrease the energy of the apparent global minimum. To maintain the template folding we used “pin” constraints. A pin allows an alpha carbon of an amino acid residue to deviate up to 1 Å from the template position without a penalty and imposed the parabolic energy penalty for larger deviations. Pins are not suitable for maintaining the folding of APHC polypeptides since these molecules are mobile in the docking procedure. Instead we applied atom-atom distance constraints to keep distances between alpha-carbons of the APHC-polypeptide close to values obtained in NMR studies. For all constraints the energy penalty was calculated using the force constant of 10 kcal∙mol^-1^∙Å^-2^.

### Data analysis and statistics

All data were expressed as the mean ± standard deviation (SD). Significance of the effect was tested by ANOVA followed by Tukey’s post hoc analysis with the p-value of 0.05.

## Results

The cell line stably expressing rat TRPV1 was used for electrophysiological and internal Ca^2+^ fluorescent recordings. Application of TRPV1 agonists (capsaicin, 2APB or low pH) evoked currents with the reversal voltage close to zero mV. Capsaicin-evoked currents were reversibly blocked by 10 μM of antagonist of TRPV1 channels capsazepine (data shown in S1A Fig). Application of the APHC1, APHC2, APHC3 (100 nM) without activating stimuli did not cause any detectable response. In Fluo-4-based intracellular calcium assay experiments, capsaicin or 2APB were used as channel agonists. Cell responses to capsaicin were fully blocked by 10 μM capsazepine (S1B Fig). In the patch-clamp experiments the responses demonstrated pronounced rundown. To take the response rundown in account, the peptide effects were measured relative to the average value of the preceding and subsequent control responses (see S2 Fig).

### Modulation of capsaicin-evoked currents

**Fig 1:**
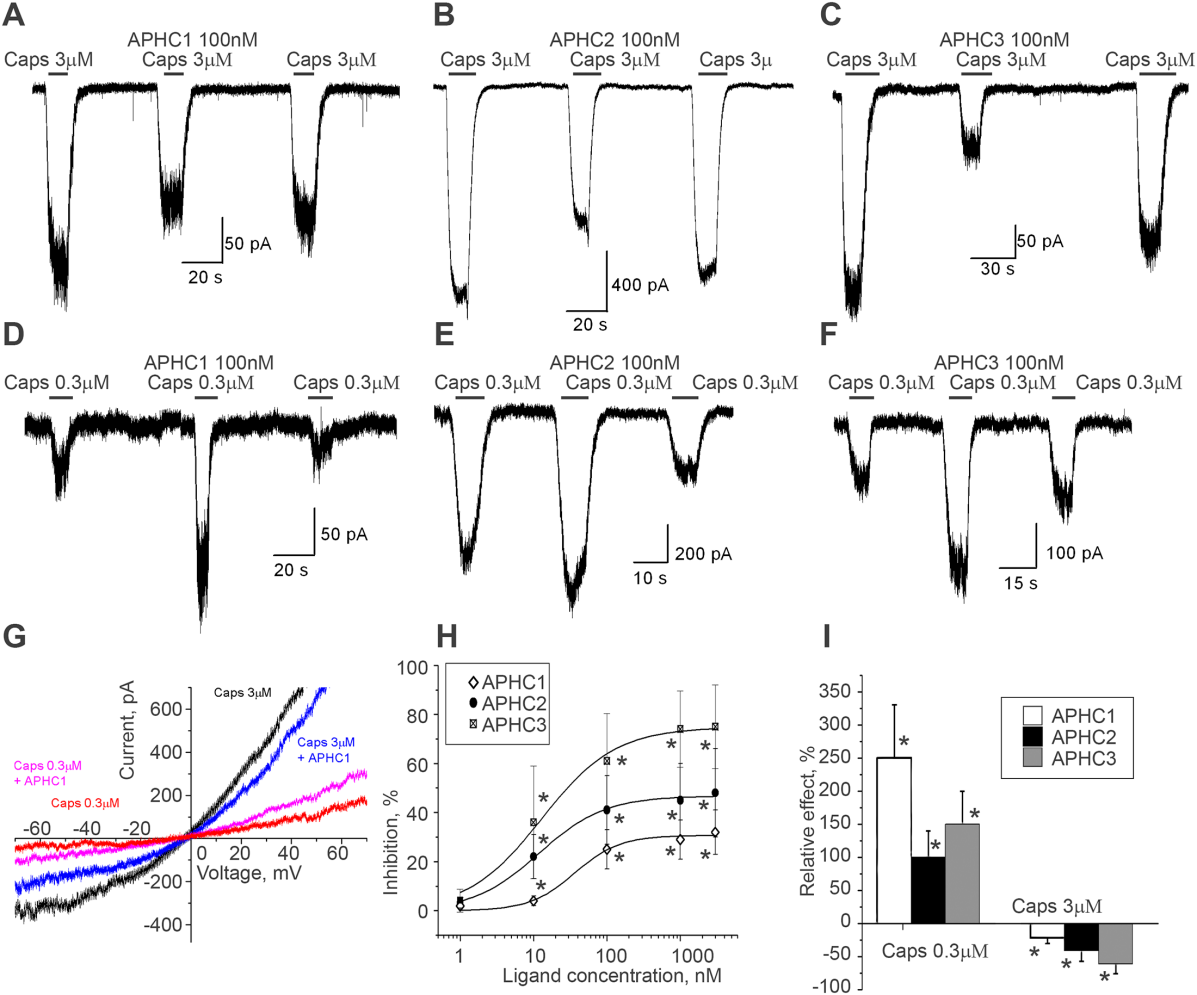
Action of APHCs on capsaicin-induced TRPV1 currents. **A-F**, Reversible inhibition or potentiation of currents evoked by 3 μM (panels A, B and C) or 0.3 μM (panels D, E and F) capsaicin by 100 nM APHC1 (panels A, D), APHC2 (panels B, E) and APHC3 (panel C, F). **G**, Current–voltage relationship for TRPV1 currents induced by 3 and 0.3 μM capsaicin in control (black and red) and in the presence of 100 nM APHC1 (blue and magenta). In both cases the toxin effect is voltage-independent. **H**, Concentration-dependence of an inhibition of TRPV1 currents activated by 3 μM capsaicin, n = 7 for each ligand. **I**, Summary of the polypeptides action (100 nM) on TRPV1 responses activated by different concentrations of capsaicin, n = 9 for all series. (*)—reliable effect by ANOVA, p = 0.05. (drug *vs* control).

Recombinant analogs of sea anemone polypeptides APHC1, APHC2, APHC3 were previously described as selective inhibitors of TRPV1 in several expression systems [23, 24]. In our present experiments, 100 nM of APHC1, APHC2 and APHC3 caused 22±8, 41±14 and 61±15% inhibition of the response caused by 3 μM of capsaicin, respectively (Fig 1A–1C). Increase of APHC concentrations up to 3 μM did not induce complete inhibition of capsaicin evoked currents. The saturation level of APHC3 effect estimated from concentration-dependence was 71±6% and IC_50_ value was 18±4 nM. Maximal inhibition for APHC2 was 42±12% and IC_50_ value was 23±9 nM (Fig 1H). The same analysis for APHC1 estimated the maximal inhibitory effect 31±9% and IC_50_ value 60 ±20 nM that were similar to a result obtained earlier for APHC1 in an electrophysiology study in oocyte expression system [24]. Thus, all polypeptides were found to be potent and incomplete inhibitors, but APHC3 induced the maximal inhibition and had the lowest IC_50_ value.

Next, we studied the action of the polypeptides on TRPV1 responses evoked by low (0.3 μM) capsaicin concentration (Fig 1D–1F). Surprisingly, responses to 0.3 μM capsaicin were markedly potentiated by all three polypeptides: 250±80%, 100±40% and 150±50% potentiation was found for 100 nM APHC1, APHC2 and APHC3 correspondingly (Fig 1D–1F). Thus, direction of APHCs action was dependent on capsaicin concentration (Fig 1I). Concentration-dependence of an inhibition of TRPV1 currents activated by 3 μM capsaicin is shown in Fig 1H. Application of 1 nM APHCs’ concentration was not effective. Effect at APHCs’ concentrations above 1000 nM reached saturation level and was not statistically differ from the one at 100 nM peptide’s concentration. Rundown of the responses together with large cell-to-cell variations did not allow to made precise estimation of IC_50_ parameters.

Analysis of the voltage-dependence of action of APHC1 was performed by 5 sec ramp protocol (Fig 1G). Percentage of current inhibition by 100 nM APHC1 decreased from 27±7% at -70 mV to 21±5% (n = 5) at +50 mV holding voltage. Potentiation of responses evoked by 0.3 μM capsaicin demonstrated the same trend. The effect decreased from 250±80% at -80 mV to 180±60% at +5 mV. However, in both cases the effect reduction at positive voltages was not statistically significant (P>0.1). The reversal potential of capsaicin evoked responses was close to zero mV for all conditions.

### Calcium assay experiments

**Fig 2:**
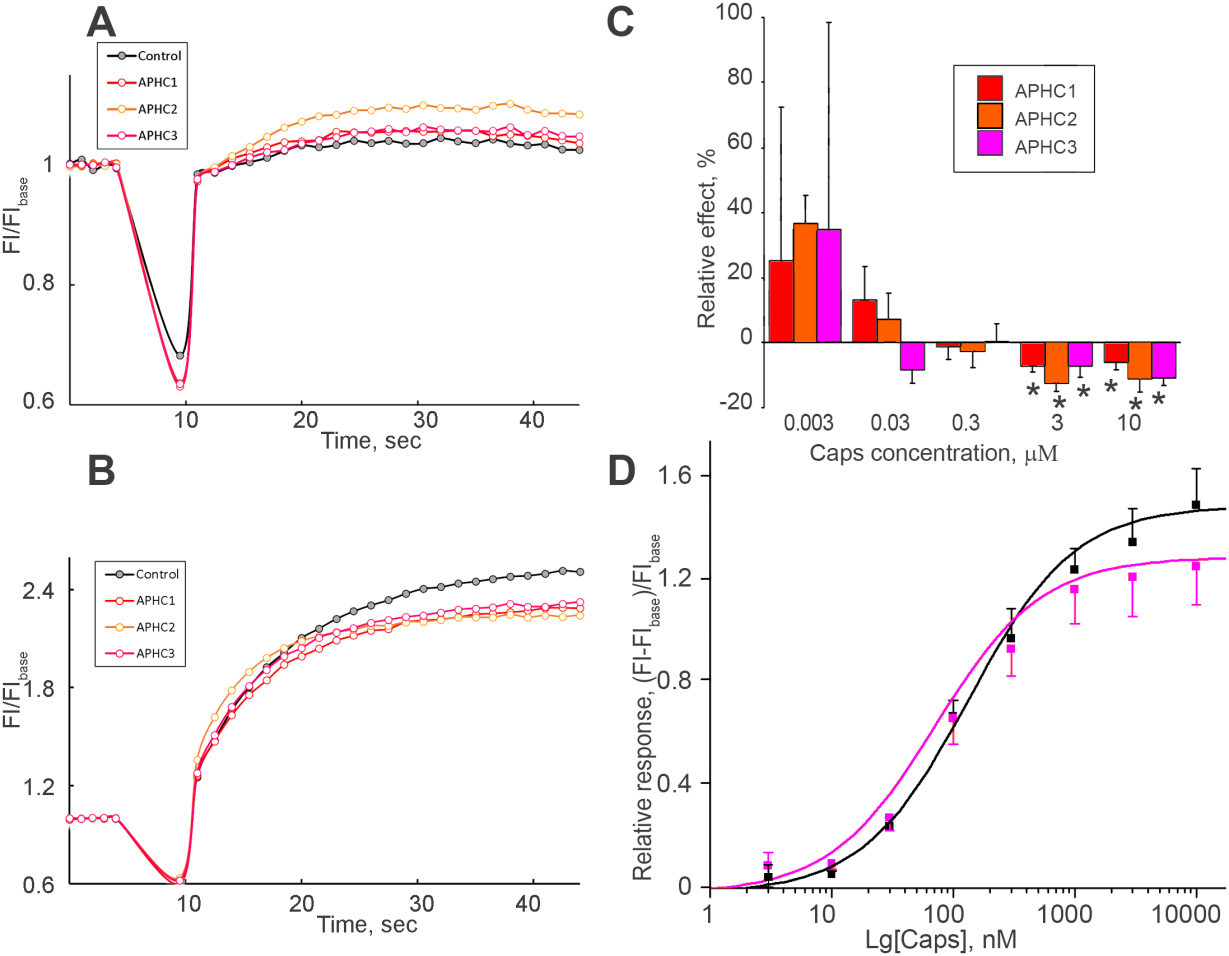
Bimodal action of APHC3 on capsaicin-evoked response of TRPV1. [Ca^2+^] responses of rTRPV1-CHO cells to 3 nM capsaicin (**A**) and 3 μM capsaicin (**B**) alone (control) and in the presence of 500 nM APHC1, APHC2, APHC3. [Ca^2+^] responses were measured as changes in fluorescence intensity before (FI_base_) and after agonist addition (FI). The data shown are representative average plots (n = 4) of the fluorescence signals against time during assays. **C**, Chart values of inhibiting/activating effects of APHC 1–3 (500 nM) relative to control in dependence on capsaicin concentration applied. In all graphs the values are given as mean ± SD of at least 5 independent experiments. Reliability (*) was checked by ANOVA followed by Tukey’s post hoc analysis, p = 0.05. **D**, “Dose-response” of TRPV1 activation by capsaicin alone (black squares) and in the presence of 500 nM APHC3 (red squares) determined in Fluo-4 based intracellular calcium assay tests. Relative responses were measured as (FI- FI_base_)/FI_base_, where FI is the measured peak fluorescence intensity after agonist addition, FI_base_—fluorescence intensity of the cells before agonist addition. Data are expressed as mean ± SD (n = 4–8).

The total calcium responses of cell population to different capsaicin concentrations were measured in control and in the presence of 500 nM of polypeptides. APHC1-3 alone did not produce any response (data not shown). All three polypeptides demonstrated potentiating activity for TRPV1 activation by 3 nM capsaicin (Fig 2A and 2C); no significant effect was found for 30 nM–1 μM capsaicin (Fig 2C) and partial inhibition was found in the case of TRPV1 activation by 3 and 10 μM capsaicin (Fig 2B and 2C). The summary plot of capsaicin-dependence of the APHC action is presented in Fig 2C. For APHC3 peptide the dose dependent potentiation was measured for 3 nM stimulus of capsaicin (S3 Fig). Small fluorescence response was detected both for control and APHCs’ treated cells, however 13–30% average potentiation by APHC3 in 65–2000 nM range was observed.

Thus, the results obtained in electrophysiological experiments on individual cells correlated with the results of Fluo-4-based intracellular calcium assay experiments, although certain quantitative differences were observed. Fig 2D presents the experimentally obtained dependence of responses in control and in the presence of 500 nM APHC3 on capsaicin concentration. Potentiation at low concentrations and inhibition at high concentrations can be interpreted as increase of capsaicin affinity and decrease of its efficacy in the presence of the toxin.

### Modulation of the response evoked by 2APB or low pH

**Fig 3:**
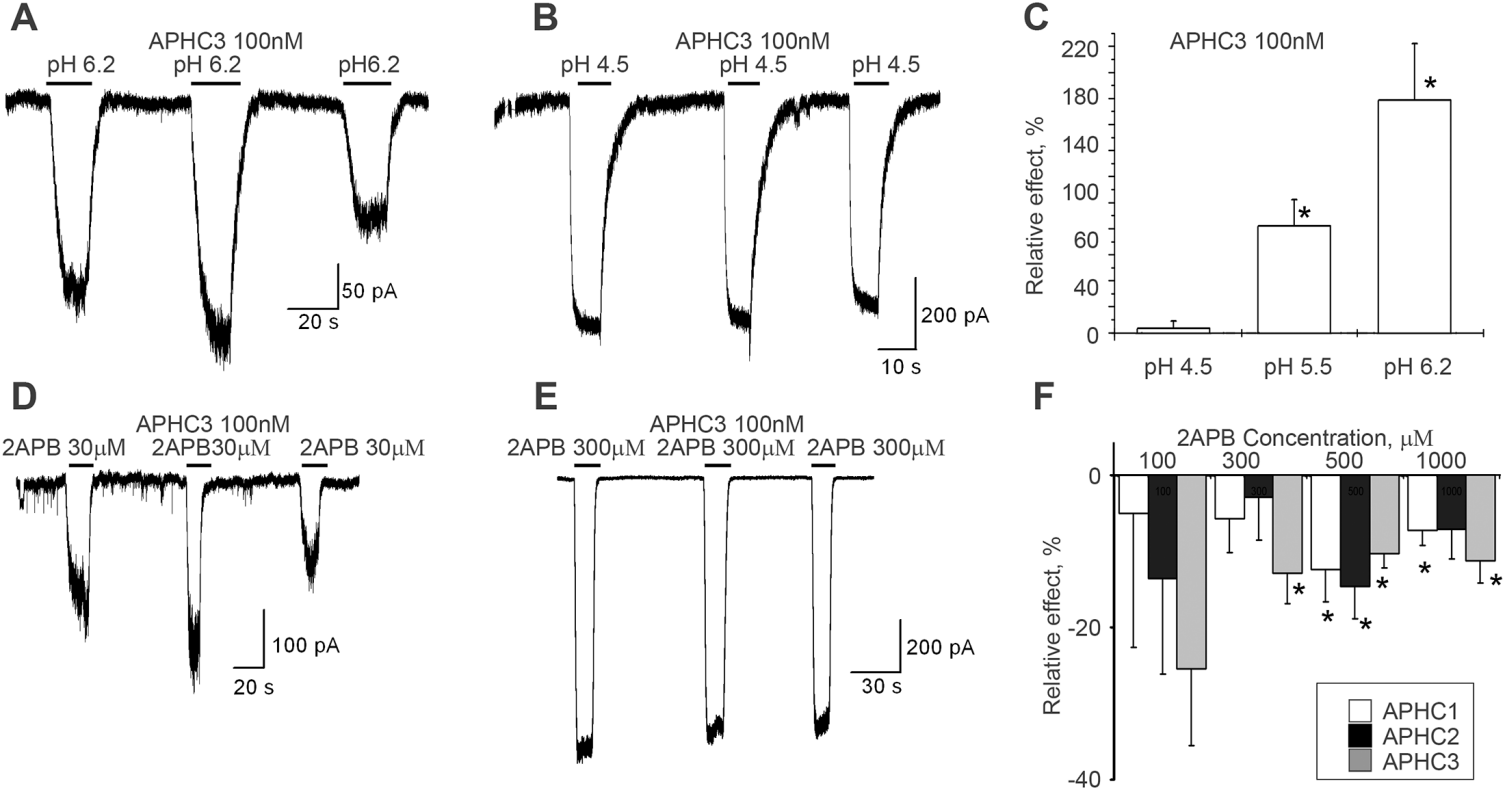
Action of APHCs on TRPV1 currents activated by 2APB or low pH stimuli. **A-C**, effect of 100 nM APHC3 on TRPV1 currents activated by pH 6.2 (potentiation) (panel A) and pH 4.5 (no effect) (panel B), traces of cell responses in control and at peptide application are shown. Panel C summarize the effect of APHC3 (100 nM) for three different pH values, n = 9 in all series. **D-E**, Effect of 100 nM APHC3 on TRPV1 currents activated by 30 μM 2APB (potentiation) (panel D) and 300 μM 2APB (no effect) (panel E), traces of cell responses in control and at peptide application are shown. **F**, Chart values of APHC 1–3 (500 nM) inhibition effects relative to control in dependence on 2APB concentration applied in Fluo-4 based intracellular calcium assay tests. In all graphs the values are given as mean ± SD of at least 5 independent experiments. Reliability drug *vs* control (*) was checked by ANOVA followed by Tukey’s post hoc analysis, p = 0.05.

Taking into account the dependence of APHCs mode of action (potentiation or inhibition) on capsaicin concentration, we used different concentrations of protons and 2APB to further characterize action of APHC1, APHC2 and APHC3. The currents evoked by drops of extracellular pH from 7.3 to 6.2 were significantly potentiated by application of 100 nM APHC2 (200±80%) and APHC3 (180±40%). Representative currents for APHC3 are presented in Fig 3A. In contrast, APHC1 did not cause significant effects on the responses evoked by the pH drop. This potentiating effect of APHC2 and APHC3 polypeptides decreased when the channel was activated by stronger acidifications. The effect of APHC3 (100 nM) was reduced to 80±20% at pH 5.5 and became non-significant at pH 4.5 (Fig 3B and 3C). APHC2 showed the same tendency.

Effects of APHCs on TRPV1 activated by different concentration of 2APB were qualitatively similar to their effect on responses evoked by pH drops. Polypeptides at 100 nM concentration potentiated the currents activated by low concentrations of 2APB (30 μM)—160±40%, 170±30% and 170±50% for APHC1, APHC2 and APHC3 correspondingly. However, currents evoked by 300 μM 2APB were not affected significantly, although certain inhibitory trend was observed (see Fig 3D and 3E for APHC3 action).

In Fluo-4-based intracellular calcium assay experiments, application of low 2APB concentrations (less than 100 μM) did not result in a detectable Ca^2+^ influx. APHC1, APHC2 and APHC3 at 500 nM showed small inhibition (generally less than 20%) in the case of channel activation by high 2APB concentrations (100 μM–1 mM) (see Fig 3F). Maximal effect (about 25±9%) was detected for APHC3 at channel activation by 100 μM 2APB.

**Fig 4:**
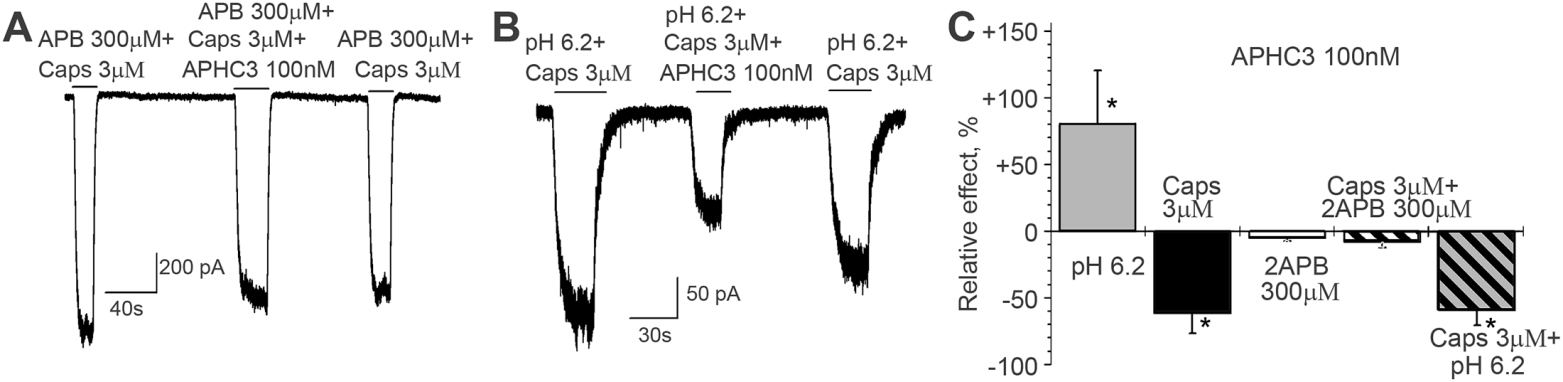
Relative effect of APHC3 on TRPV1 currents in response to single and multiple stimuli activation. Effect of 100 nM APHC3 on TRPV1 currents evoked by co-application of capsaicin and 2APB (**A**), or capsaicin and low pH (**B**), traces of cell responses in control and at peptide application are shown. **C**, Summary of the data. The values are given as mean ± SD. Reliability to control (*) was checked by ANOVA followed by Tukey’s post hoc analysis, p = 0.05. Effect on capsaicin-evoked response (n = 9) to co-application of capsaicin and low pH (n = 6) was not statistically different. Effect on 2APB alone (n = 7) to 2APB plus capsaicin (n = 6) was not statistically different.

Since APHC3 polypeptide was the most active, we studied its action on the TRPV1 activated by co-application of capsaicin with 2APB or with low pH. The results shown in Fig 4 demonstrate that action of 100 nM APHC3 on response evoked by 3 μM capsaicin together with 0.3 mM 2APB is similar to that response evoked by 2APB alone. In both cases the effect of APHC3 was not statistically significant. The inhibitory action of APHC3 on channel activation by 3 μM capsaicin with low pH was approximately equal to the polypeptide effect at channel activation by capsaicin alone, contrary to APHC3 potentiating action at application of pH 6.2 alone. Thus, effects of the APHCs on the response activated by combinations of different stimuli are non-additive.

### Kinetics of APHC action

**Fig 5:**
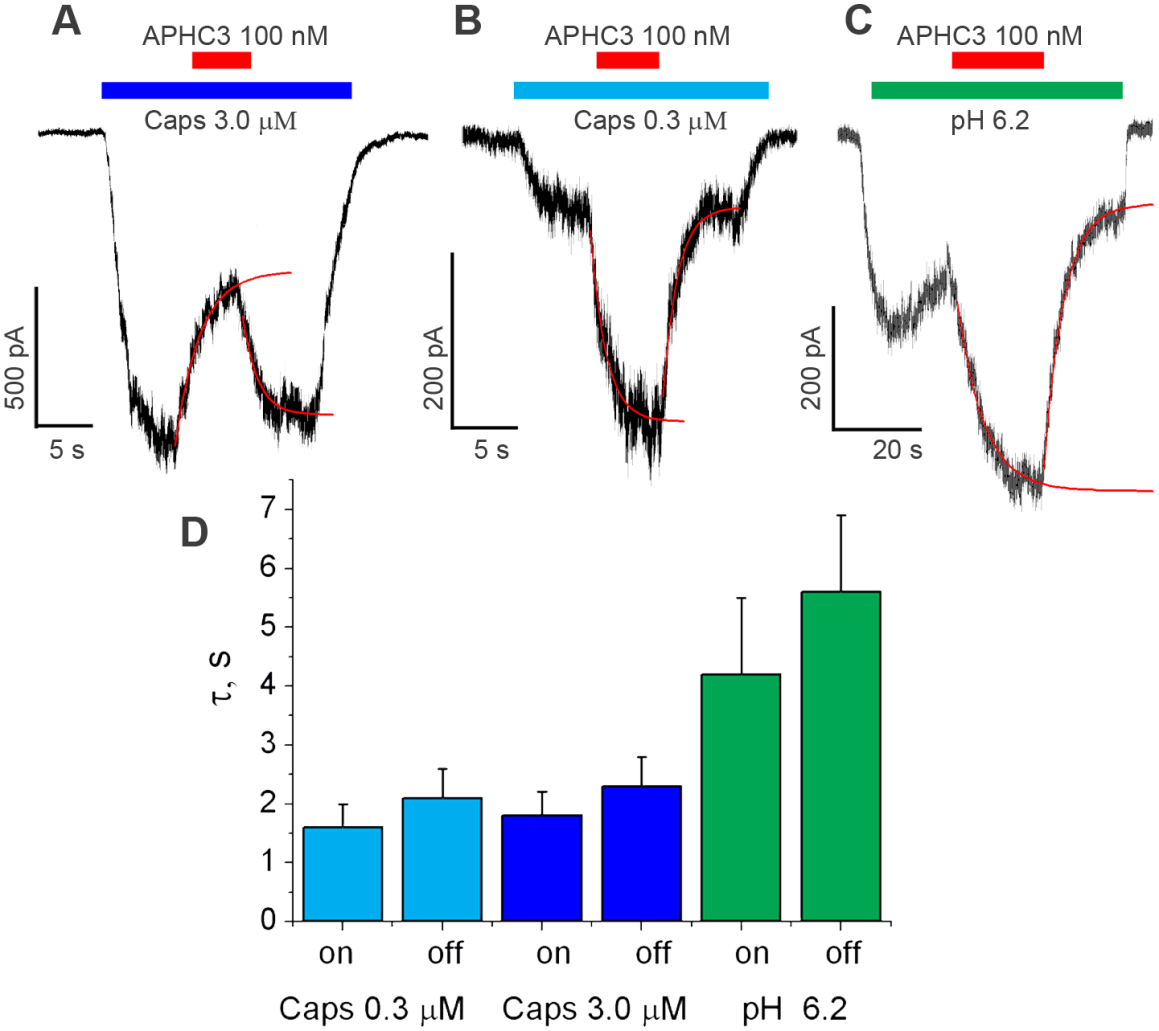
Kinetics of 100 nM APHC3 action in the continuous presence of activation stimuli. **A**, Reversible inhibition of the current evoked by 3 μM capsaicin. **B**, Reversible potentiation of the current evoked by 0.3 μM capsaicin. **C**, Reversible potentiation of the current evoked by pH drop to 6.2. Single-exponential fitting in panels A-C are shown as red curves. **D**, Averaged values of time constants of APHC3 effects development and recovery (n = 5 in all series). The kinetics of action is significantly slower in the case of TRPV1 activation by pH drop.

Existence of two opposite effects (potentiation and inhibition) raises the question about site(s) of action. Indeed, the effects could be induced by complex allosteric interaction with a single binding site or by independent binding to distinct sites. To check a possibility of APHC binding to distinct sites we studied kinetics of action in the presence of capsaicin. Fig 5 demonstrates the representative results. APHC3 effects on the currents evoked by low (Fig 5A) and high (Fig 5B) capsaicin concentrations as well as potentiation of the proton evoked current (Fig 5C) developed monotonously and were well-fitted by single-exponential function. Development of the effects was much slower than the overall speed of the application system (see S4 Fig). Recovery from the effects was also monotonous and fast in all cases suggesting that APHC3 does not significantly affect TRPV1 desensitization.

Moreover, kinetics of potentiation and inhibition of the capsaicin-evoked responses did not differ significantly (Fig 5D). It is unlikely that two independent effects mediated by APHC binding to distinct sites can demonstrate matching kinetics. Thus, our data suggest that inhibition and potentiation of capsaicin-evoked responses are caused by APHC binding to the single site. The kinetics of APHC3 action on proton-evoked currents was significantly slower (P<0.05). The time constants were about 2 sec in the case of capsaicin-evoked responses and were more than 4 sec for proton-evoked currents (Fig 5D).

### Molecular modeling

**Fig 6:**
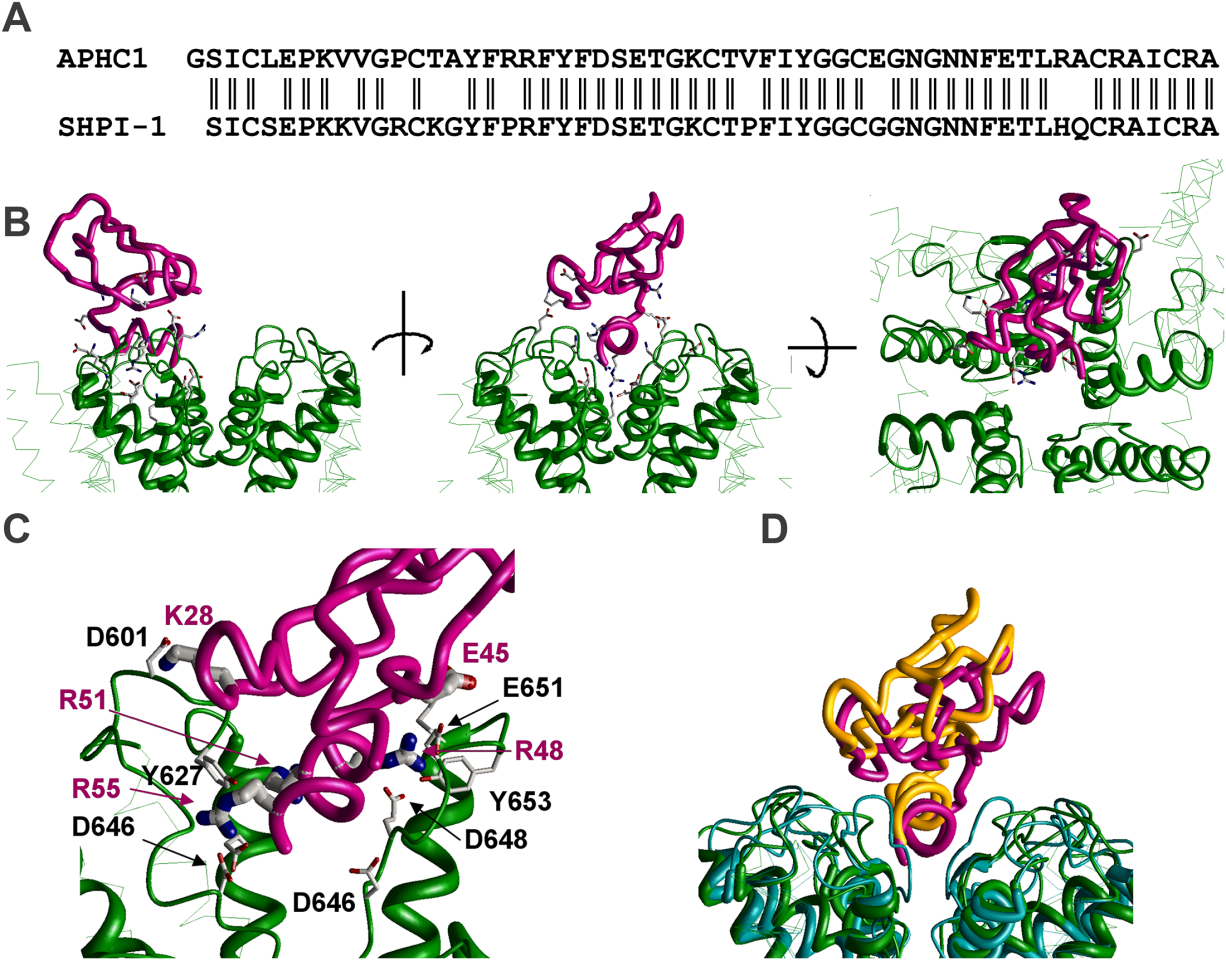
The model of APHC1 binding with TRPV1 outer region. **A**, Aligned sequences of APHC1 and trypsin inhibitor SHPI-1. **B**, Different views on the minimal-energy complex calculated for APHC1 and close stated TRPV1. Backbone of APHC1 is shown magenta, backbone of the channel is colored green. The *C*-terminal helix of the APHC1 intercalates between diverging P-loops. **C**, The details of polypeptide-channel interaction. Key residues involved in the interaction are shown as sticks. **D**, Superimposition of two APHC1 binding models—APHC1—closed TRPV1 (green and magenta) and APHC1—opened TRPV1 (cyan and orange). In the open state the slot between P-loops is narrower and APHC1 is shifted in the external direction.

To reveal possible binding site of the APHC-polypeptides and predominant structural determinants of their action we employed the molecular modeling approach. This became possible due to a recent resolution of the atomic-scale structure of rat TRPV1 in different functional states [28]. Since 3D structures of the APHC-polypeptides are unavailable we used a model based on the structure of highly homologous trypsin inhibitor from sea anemone *S*. *helianthus* SHPI-1 (PDB code 1SHP) (Fig 6A). We docked APHC1 to the closed TRPV1 structure (PDB code 3J5P) using a two-stage protocol. At the first stage 30,000 random positions and orientations of the APHC1 in the vicinity of the TRPV1 outer pore were generated. Each structure was shortly optimized to remove steric clashes. For the next stage of docking we selected 100 best-energy structures. These structures were optimized until 1000 consecutive energy minimization did not improve the complex energy. For the minimal-energy complex (Fig 6B) the key interacting pairs of residues were determined (Fig 6C).

In this optimal binding mode, the *C*-terminal helix of the APHC1 is incorporated between P-loops of two adjacent subunits. APHC1 interacts significantly with the pore helix and two external loops of TRPV1, which are involved in the extracellular gating and undergo significant conformational rearrangements. The strongest toxin-channel interactions in the model are provided by positively charged Arg residues in the *C*-terminal helix (positions 48, 51 and 55). Arg48 interacts with Glu648, Glu651 and Tyr653. Arg51 interacts with Glu636 and Tyr627 of another subunit. The terminal Arg55 interacts with Asp646 of this subunit. There are also significant interactions between Lys28 of the toxin and Asp601 of the channel and between Glu45 and Lys656. These interacting residues are shown in Fig 6C. Thus, strong interactions were found in the model between the toxin molecule and adjacent subunits of TRPV1.

To reveal possible determinants of the activity-dependent action of APHCs we performed the docking procedure for the TRPV1 open state model (PDB code 3J5Q). The superimposition of two minimal-energy binding models for the channel open and close states (Fig 6D) showed similar interactions in both cases. However, since the loop between pore helix and S6 segment has different conformations in the open and close state, the slot, which accommodates the *C*-helix of APHC1, is narrower in the open state than in the closed state. As a result, in the open state APHC1 is pushed about 2.5 Å in the external direction (Fig 6D). Thus, conformation rearrangements associated with external gating change the binding position of the APHCs and *vice versa*; APHCs binding can affect equilibrium between close, intermediate and open states.

## Discussion

The unique properties of TRPV1, as a sensor and an integrator of different stimuli, make it a very exciting target for the study and treatment of pain as well as other pathological states [36, 37]. The usual directions of drug development searching for potent agonists and antagonists of TRPV1 were significantly discredited because of the side effects of new compounds [36]. On the other hand, the multimodality of TRPV1 activation provides additional opportunities for drug design [38]. Therefore, understanding the mechanisms underlying the effects of partial antagonists is an important step to developing effective drugs affecting TRPV1 function.

Usually, considering the ligand-receptor interaction implies that the ligand can induce stable and invariable effects on the receptor—activation, potentiation or inhibition. In the present work we aimed to characterize action of recently discovered sea anemone peptides APHC1-3 on the TRPV1 channels. The main finding of our work is that action of these polypeptides depends not only on the nature of activation stimuli, which is known for many TRPV ligands, but also on the strength of the stimuli. At low activation-strength stimuli, all APHC-polypeptides mainly potentiated TRPV1 response while at increasing activation strength the potentiating effects disappeared or switched to inhibition in the case of activation by capsaicin.

Although both electrophysiological and Ca^2+^ assay approaches have revealed activity-dependence of the APHCs action, some quantitative details do not coincide. For example, the range of agonist concentrations resulted to potentiation or inhibition effect mismatched for electrophysiological recordings and fluorescent Ca^2+^ measurements. Activation of TRPV1 by 0.3 μM capsaicin in the presence of APHCs has significant potentiation effect in electrophysiological experiments (Fig 1D–1F) while at the same capsaicin concentration in calcium assay experiments (Fig 2D) the effect was close to zero, and potentiating was observed at lower agonist concentrations. Also it should be emphasized that APHC3 produce unequal effects on pH activation in different experimental conditions; it could either potentiate pH response in electrophysiological experiments (this paper) or inhibit it in single cell Ca^2+^ imaging [23]. Such dissimilarities could occur due to differences in the experiment conditions. For example, in patch clamp experiments the membrane voltage is kept constant, whereas in calcium measurements activation of TRPV1 and their modulation by polypeptides obviously affect the voltage and thus the calcium influx. As a result of this negative feedback the results of calcium measurements are not proportional to the conductance. From the other side, voltage clamping is artificial approach and results of calcium measurements may be more relevant to physiological conditions.

Important question is to discriminate direct effect of a drug or toxin and indirect influence mediated by secondary messengers. For instance, it has been shown that Gigantoxin I isolated form sea anemone potentiates TRPV1 by modulation of the PLA2 pathway [39]. From the other side, a bivalent tarantula toxin activates TRPV1 by direct action [21]. We cannot rule out a possibility of indirect action but our data favor direct mechanism. First, kinetics of APHCs action is rather fast. Potentiation and inhibition develop approximately as fast as response to capsaicin or the pH drop. Recovery from the APHC action also takes place in timescale of seconds (see Fig 5). Second, in the whole-cell patch clamp experiments the intracellular content is largely dissociated and involvement of intracellular messengers in the modulation is unlikely. Therefore we concentrated on the putative mechanisms of direct action.

Mechanism of APHCs action is complex. Our data suggest that in the presence of APHCs the agonist apparent affinity is increased but the efficacy is decreased. It means stabilization of some intermediate non-conducting state of the channel. Rationalization of these data is possible in view of recently proposed double-gate scheme of TRPV1 activation, which is based on atomic-scale structures of TRPV1 [28, 29]. In this scheme, two distinct gate regions recognized by cryo electron microscopy [28] suggest four principal states of the receptor. In the absence of activating stimuli both gates are closed. Binding of an activating ligand causes sequential opening of both gates, which are coupled allosterically. According to the results of our modeling calculations, APHCs bind to the external region involved in the proton activation and can directly affect opening of the external gate of TRPV1. This can promote allosteric opening of external gate after capsaicin binding and can cause potentiation of proton binding. The mechanism of decreasing of apparent capsaicin efficacy revealed in our experiments is unclear. There are several possibilities, which cannot be discriminated at present. First, positively charged toxin residues in our model occurs rather close to the permeation pathway and can cause a reduction of channel conductance. Second possibility is that open-time or fast desensitization characteristics of the external gate are affected by the toxin binding. The third possibility is that allosteric coupling between external and internal gates is affected by APHCs that results in stabilization of an intermediate non-conductive state in the double-gate scheme of activation. Further studies are required to resolve these uncertainties.

Modulators of TRPV1 activation can produce positive therapeutic effects without common side effects such as hyperthermia. Response modulation to low pH stimuli is considered an important factor determining the hyperthermia effect of the TRPV1 antagonists *in vivo*. Antagonists that are able to inhibit pH-induced TRPV1 currents elicit a hyperthermic effect, while potentiation of the pH-induced activation of TRPV1 either decrease or do not change body temperature [8, 40]. APHC1 did not produce significant effects on pH activation of TRPV1 and caused significant decrease of body temperature *in vivo* tests [23]. APHC3 is able to produce dissimilar effect on pH activation from potentiation (this study) to inhibition and this result in moderate decrease of core body temperature [23]. It should be emphasized that APHC3 produce dissimilar effects on pH activation in the different experimental conditions; it could either potentiate pH response in electrophysiological experiments (this paper) or inhibit it in single cell Ca^2+^ imaging [23].

Thus reliable prediction of *in vivo* effects of APHC polypeptides cannot be made at present time taking into account the complexity of physiological processes associated with TRPV1 and the complexity of polypeptides action demonstrated above. In particular, we showed that action of APHC3 on the TRPV1 currents evoked by a mixture of capsaicin and 2APB or capsaicin and acidic pH is not a simple sum of its effects on the currents evoked by individual stimuli. Nevertheless, modulators with dualistic effects may have a distinct advantage in terms of their practical medical application. Such drug compounds don’t inhibit normal receptor function. Only in the case of pathologically strong activation the desired therapeutic effect will occur.

## Supporting information

S1 Fig Characterization of cell line stably expressing rat TRPV1 by electrophysiological (A) and intracellular calcium assay (B) experiments. Selective TRPV1 agonist capsaicin was applied for the channel activation that was abolished by 10 μM capsazepine almost completely.
(TIF)

S2 Fig Analysis of APHCs action on the capsaicin evoked responses. To compensate a significant response rundown effects of polypeptides were measured relative to the average amplitude of the preceding and subsequent control responses of capsaicin application.
(TIF)

S3 Fig Dose-response dependence for APHC3 potentiation on (Ca^2+^) response induced by 3 nM capsaicin in CHO cells expressing rTRPV1. Relative responses were measured as (FI- FI_base_)/FI_base_, where FI is the measured peak fluorescence intensity, FI_base_ is the fluorescence intensity in cells before capsaicin addition (n = 4). Potentiation is expressed as percentage of the response in control experiments (untreated by APHC3 cells). Bars show the average value of 9 independent experiments which are presented in different colors.
(TIF)

S4 Fig Representative whole-cell recording shows that application of high-potassium external solution is rather fast. The new equilibrium level of holding current can be reached during 100–200 ms. Development of capsaicin or drug effect was much slower (see Fig 5).
(TIF)

S1 File Raw data file. (ZIP)
