# Supplementary figures and images for: TRPV1 activation power can switch an action mode for its polypeptide ligands
Source: PLoS One. 2017 May 5;12(5):e0177077. doi: 10.1371/journal.pone.0177077 (PMC5419573; doi:10.1371/journal.pone.0177077)

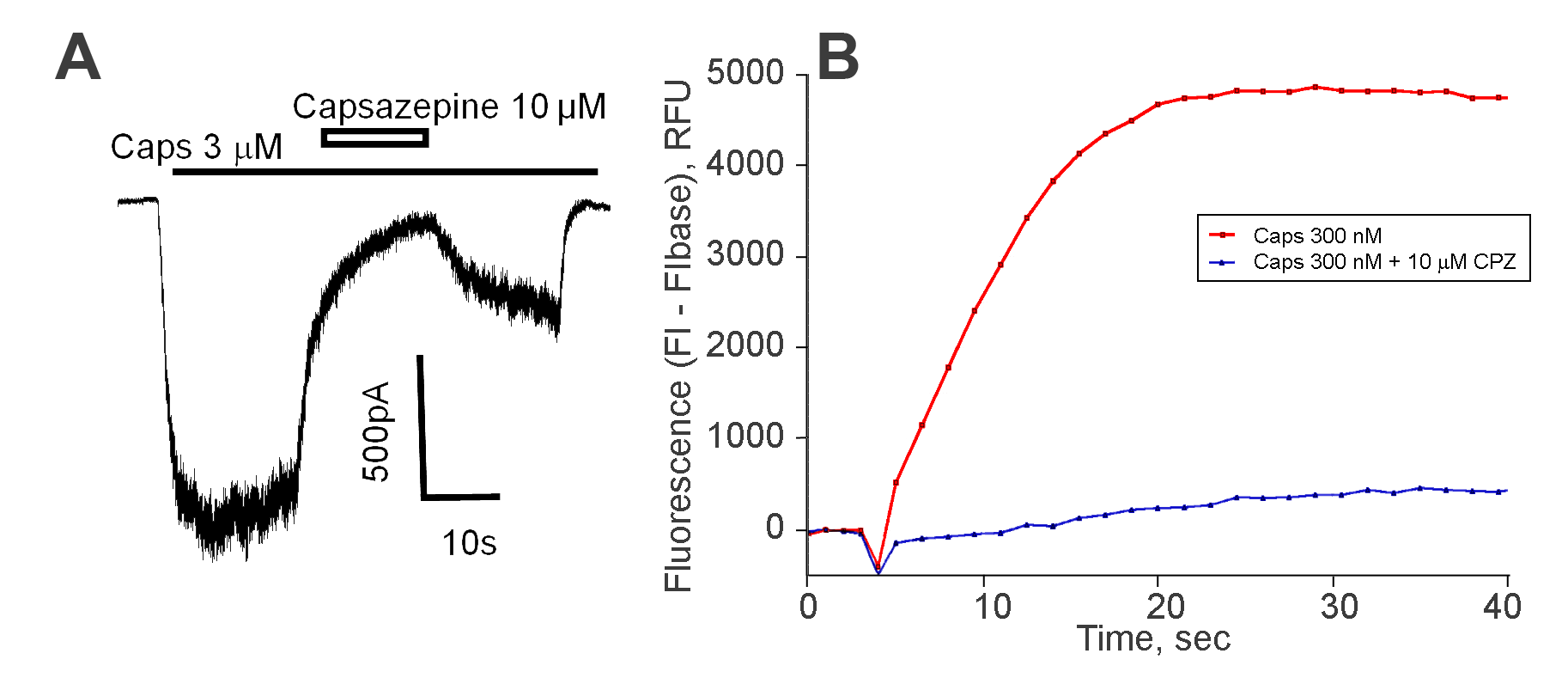

Supplement: S1 Fig — Selective TRPV1 agonist capsaicin was applied for the channel activation that was abolished by 10 μM capsazepine almost completely. (TIF) [file pone.0177077.s001.tif]

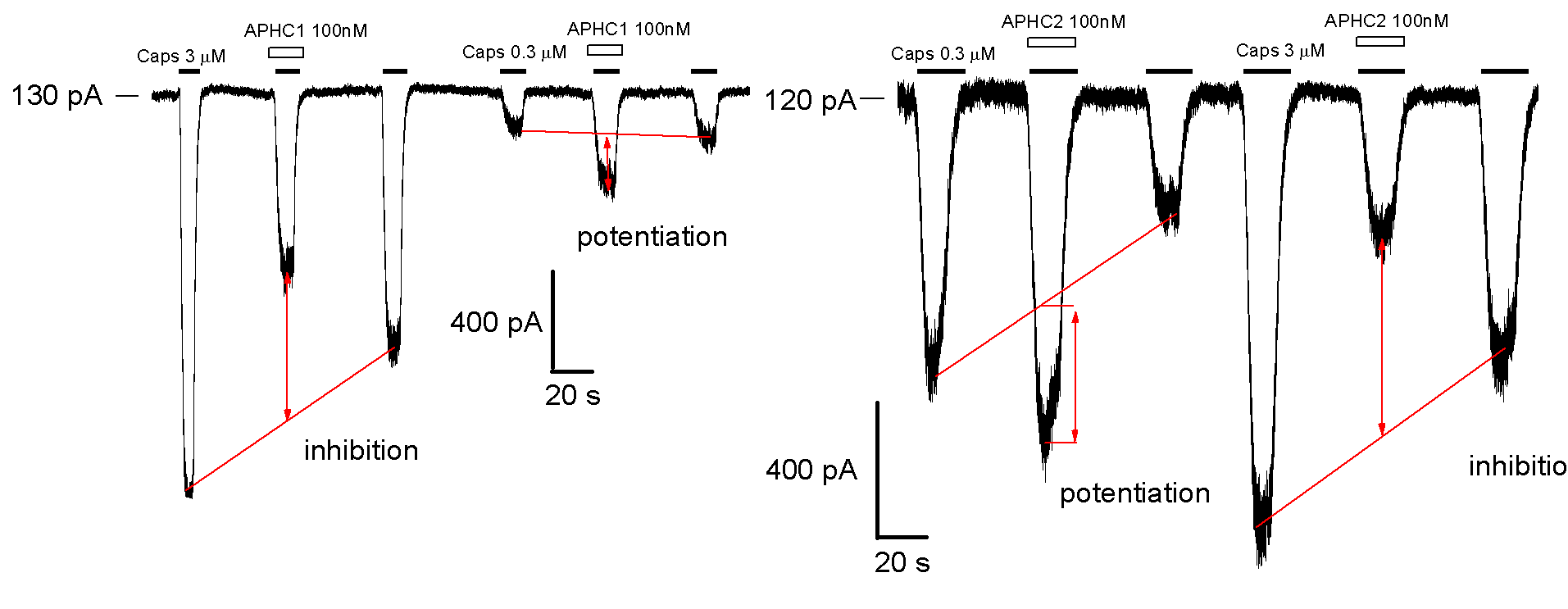

Supplement: S2 Fig — To compensate a significant response rundown effects of polypeptides were measured relative to the average amplitude of the preceding and subsequent control responses of capsaicin application. (TIF) [file pone.0177077.s002.tif]

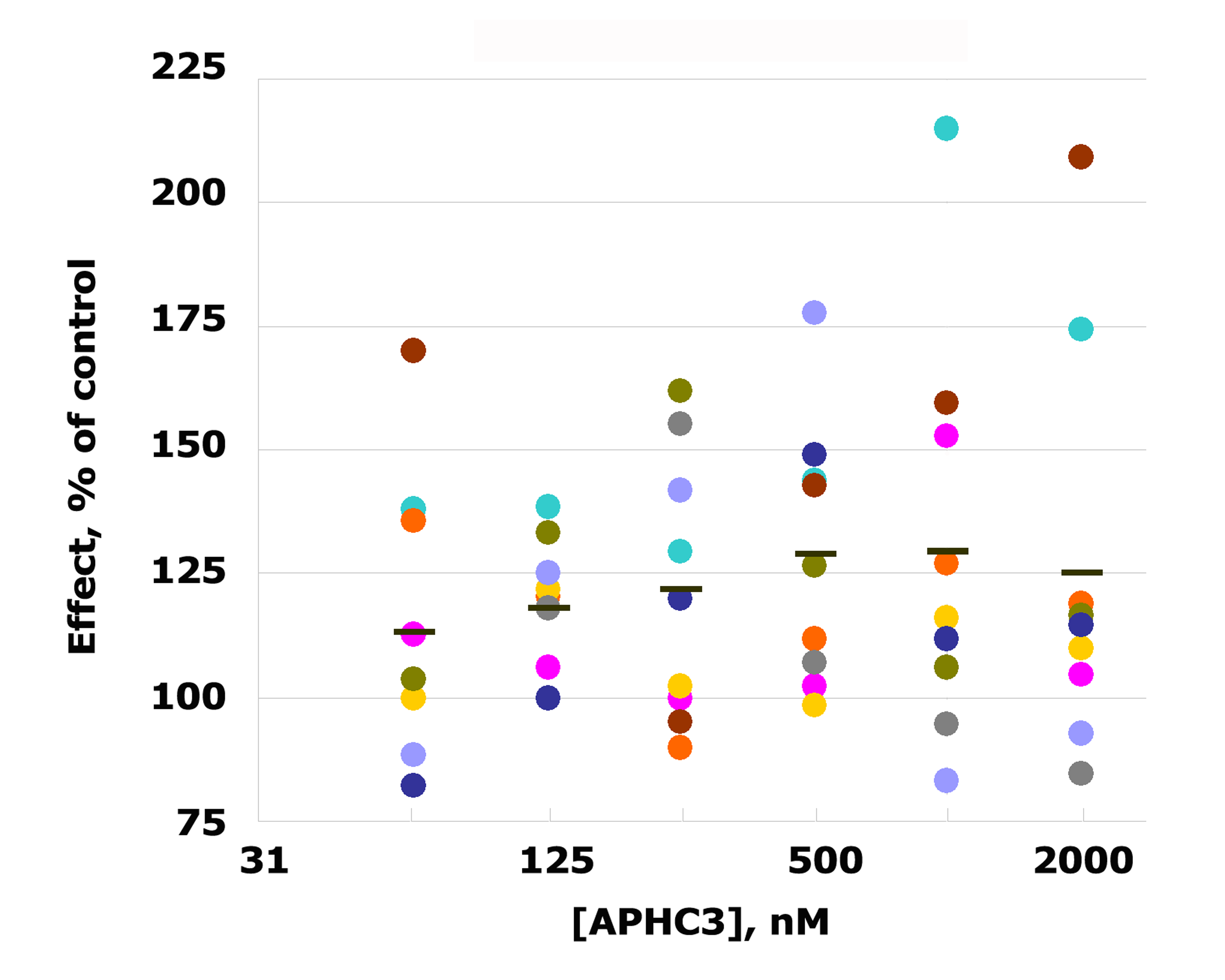

Supplement: S3 Fig — Relative responses were measured as (FI- FIbase)/FIbase, where FI is the measured peak fluorescence intensity, FIbase is the fluorescence intensity in cells before capsaicin addition (n = 4). Potentiation is expressed as percentage of the response in control experiments (untreated by APHC3 cells). Bars show the average value of 9 independent experiments which are presented in different colors. (TIF) [file pone.0177077.s003.tif]

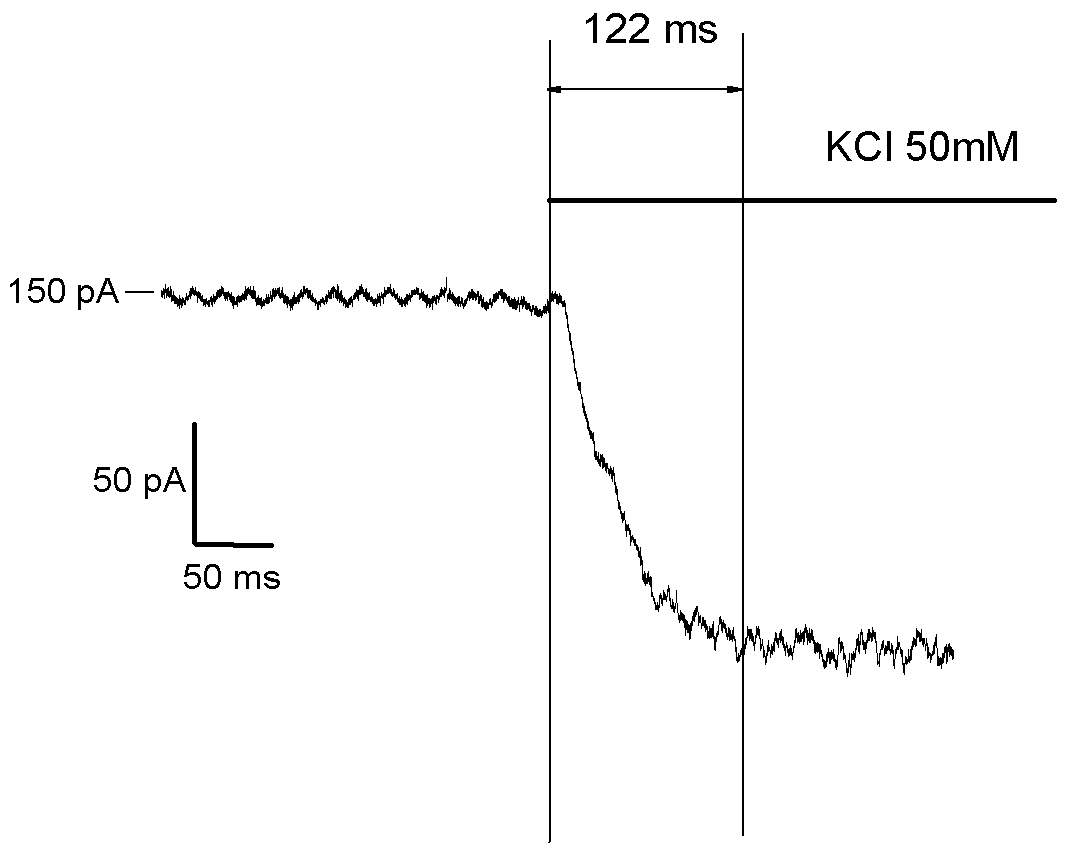

Supplement: S4 Fig — The new equilibrium level of holding current can be reached during 100–200 ms. Development of capsaicin or drug effect was much slower (see Fig 5). (TIF) [file pone.0177077.s004.tif]
